# Progression of Skin Ulcer Secondary to Calciphylaxis in a Patient With End-Stage Renal Disease (ESRD) on Hemodialysis and the Therapeutic Approach

**DOI:** 10.7759/cureus.55161

**Published:** 2024-02-28

**Authors:** Yamama Al-Khazraji, Mina Al-Khazraji, Oladimeji Oluaderounmu, Bryan Quintanilla

**Affiliations:** 1 Internal Medicine, Metropolitan Hospital Center, New York City, USA; 2 Internal Medicine, Royal Bolton Hospital, Bolton, GBR

**Keywords:** end-stage renal disease (esrd), hemodialysis related, painful skin lesions, warfarin-induced skin necrosis, treatment of calciphylaxis

## Abstract

Calciphylaxis is a rare and severe medical condition characterized by the calcification of small blood vessels and soft tissues, leading to tissue damage, skin ulcers, and intense pain. It most commonly affects individuals with underlying health conditions such as kidney disease, particularly end-stage renal disease (ESRD), and is associated with high mortality rates. Understanding the diagnosis and management of calciphylaxis is crucial for improving patient outcomes. Diagnosing calciphylaxis can be challenging due to its rarity and overlapping symptoms with other skin conditions. Healthcare professionals typically use a combination of clinical evaluation and diagnostic tests to reach a conclusive diagnosis.

The management of calciphylaxis is multifaceted and typically involves a collaborative effort between various healthcare specialists, including nephrologists, dermatologists, and wound care experts. The primary goals of treatment are to alleviate pain, promote wound healing, address underlying causes, and prevent further complications.

## Introduction

Calciphylaxis is a severe and life-threatening arteriolopathy defined as vascular calcification resulting in the occlusion of microvessels in the subcutaneous adipose tissue and dermis, creating intensely painful, ischemic skin lesions [1,2]. It often occurs in patients with hyperparathyroidism linked to end-stage renal failure and patients undergoing hemodialysis (HD) [3]. The typical clinical picture is of an indurated skin lesion resembling livedo reticularis on the lower limbs, which may progress to violaceous, painful, plaque or subcutaneous nodules, followed by ischemic/necrotic ulcers of reticular pattern with or without Eschars [4,5].

## Case presentation

**Figure 1:**
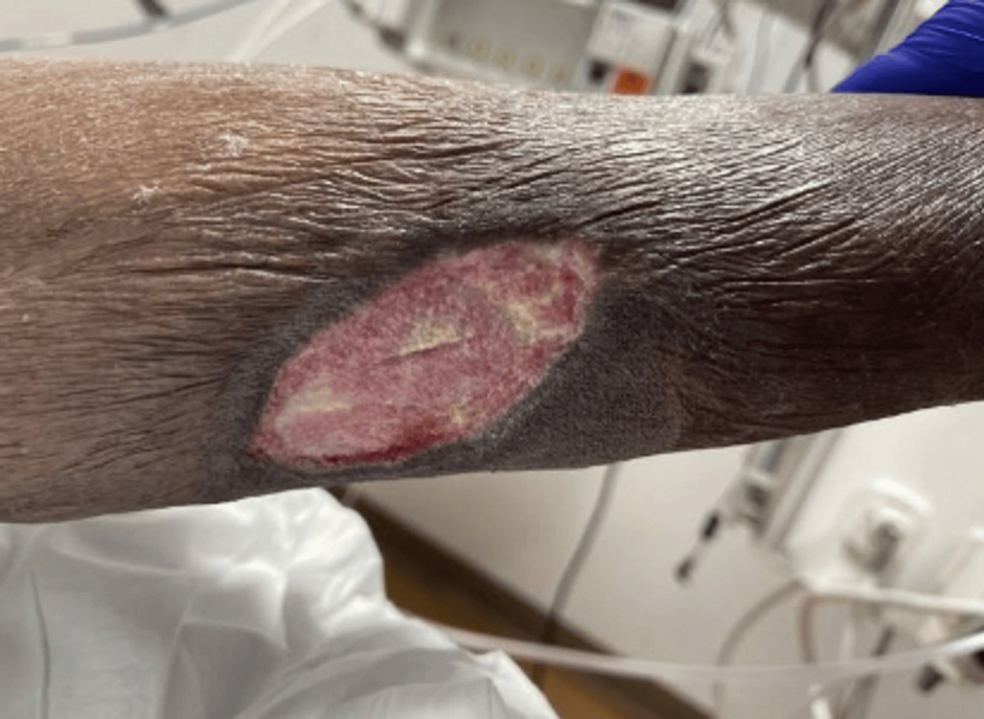
Left leg ulcer.

A 62-year-old African American woman with a history of end-stage renal disease (ESRD) on hemodialysis (HD) due to ESRD following lupus nephritis., antiphospholipid syndrome, hypertension, chronic obstructive pulmonary disease, recurrent deep venous thrombosis on Warfarin, presented with progressive leg pain that was associated with a painful necrotic lesion on the left leg about 4*7 cm in diameter surrounded by erythema with a foul smell (Figure 1). The pain was severe enough to limit her ability to ambulate. There was no associated fever, chills, chest pain, or dyspnea.

Her home medications and laboratory findings are in Tables 1, 2, respectively.

**Table 1:** Home medications of the patient.

Home medications
Mycophenolate 360 mg twice daily
Hydroxychloroquine 200 mg daily
Warfarin 2.5 mg daily
Amlodipine 10 mg daily
Lisinopril 40 mg daily
Pantoprazole 40 mg daily
Clopidogrel 75 mg daily
Sertraline 25 mg daily
Atorvastatin 40 mg daily
Carvedilol 6.25 mg twice daily
Ergocalciferol 50000 IU weekly
Fluticasone -Salmeterol 250-50 mg inhaler daily
Folic acid 1 mg daily
Gabapentin 300 mg daily
Ipratropium 0.02 nebulizer
Levothyroxine 25 mcg daily

**Table 2:** Laboratory findings.

Laboratory values	Result	Reference value
Blood urea nitrogen	33 mg/dL	7-21 mg/dL
Potassium	5 mmol/L	3.5-4.7 mmol/L
Calcium	7.4 mg/dL	8.5-10.1 mg/dL
Phosphorous	7.8 mg/dL	2.5-4.9 mg/dL
Parathyroid hormone ( PTH)	478 pg/mL	18.4-80.1 pg/mL
Albumin	2 g/dL	3.4-5.0 g/dL
White cell count	14.52 K/μL	4.0-11.0 K/μL
Hemoglobin	6.3 g/dL	13.0-17.0 g/dL
Erythrocyte sedimentation rate (ESR)	>150 mm/hr	0-20 mm/hr
C reactive protein (CRP)	20.9 mg/dL	0.3- 1.0 mg/dL
Cardiolipin antibodies	Positive	

The patient underwent a computed tomography (CT) scan of the lower extremity, which revealed superficial ulceration with no evidence of osteomyelitis and suspicion of subcutaneous gas-forming infection. The patient was treated with ampicillin-sulbactam and vancomycin for ten days with no clinical improvement. Skin biopsy of the ulcer showed increased uptake of Von Kossa stain for calcium, confirming the diagnosis of calciphylaxis.

Treatment with thrice weekly sodium thiosulfate 12.5 g during dialysis was initiated. During hospitalization, debridement and negative pressure wound therapy were performed for the ulcer. Additionally, cinacalcet 30 mg daily was administered to reduce her blood serum calcium and phosphate levels.

**Figure 2:**
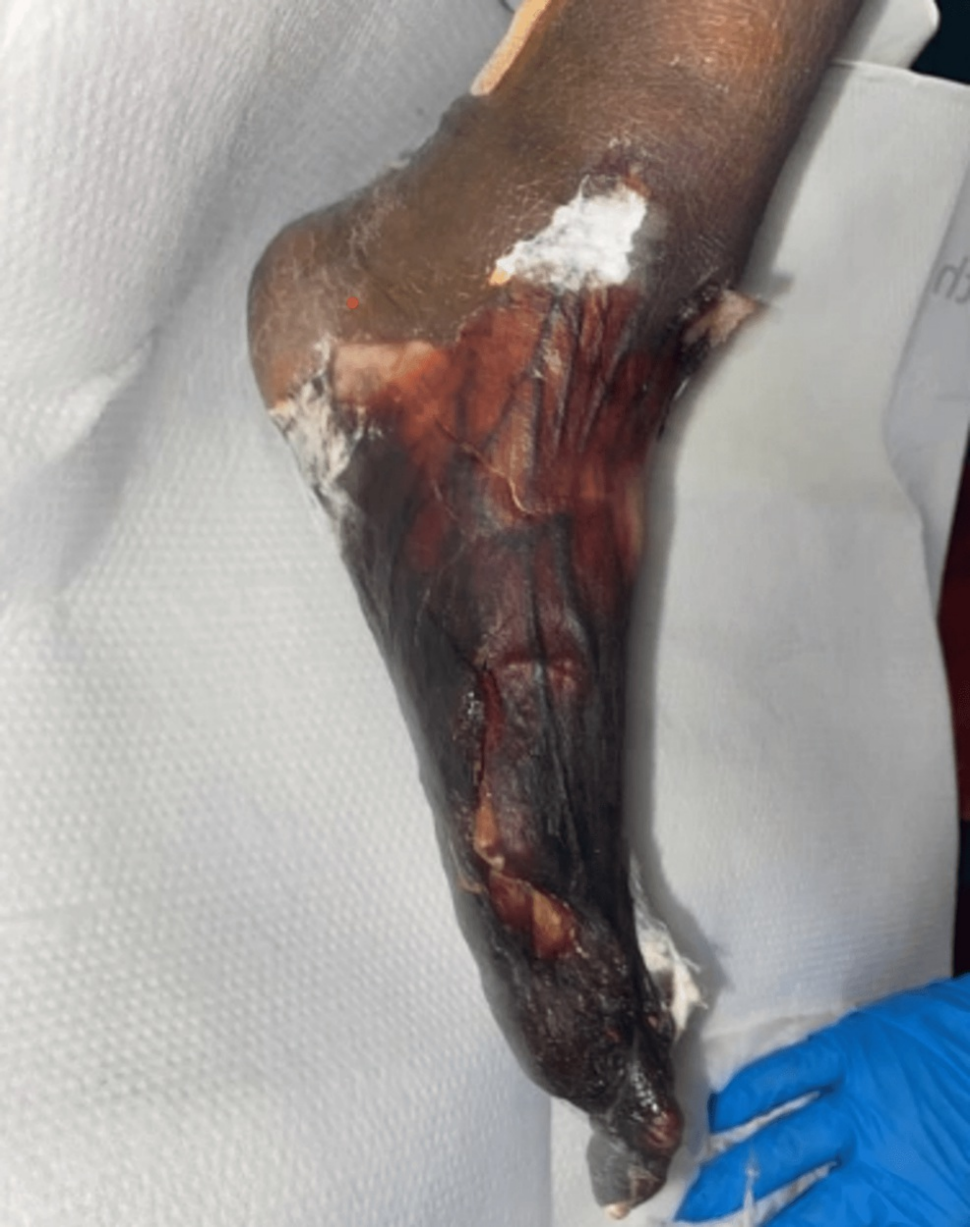
Left foot ulcer.

Three months later, she was hospitalized due to sepsis secondary to polymicrobial necrotizing fasciitis with signs of gas gangrene in the left big toe. Debridement and first toe resection were done, and the pathology report confirmed ischemic necrosis without osteomyelitis. The patient showed no improvement in three months of treatment with sodium thiosulfate - a new ulcer formed on the sacrum and the left foot (Figure 2). After extensive discussion, the patient opted for home hospice and palliative care with no more HD sessions.

## Discussion

Calciphylaxis is a devastating occlusive blood vessel disease that results from the narrowing of the arterial lumen by calcification in the media and fibrosis underneath the intima [6]. The one-year mortality rate is 50%, and it goes up to 80% in ulcerated lesions, with sepsis the most common cause of death [7,8].

Our patient had several predisposing factors for calciphylaxis, which are ESRD, hyperparathyroidism, SLE, short bowel syndrome, and warfarin intake. Evidence suggests that warfarin has been implicated in the process of vascular calcification by inhibiting vitamin K-dependent proteins that prevent calcium depositions in arteries [9,10]. This patient was on warfarin when she had her first necrotic ulcer; warfarin was discontinued soon after admission; however, the patient continued to develop new ulcers.

Cutaneous manifestations of calciphylaxis are described as livedo reticularis-like plaques which may eventually progress to soft tissue ulceration, necrosis, and non-healing eschars. The diminished blood supply puts them at risk of infection. The most affected sites are the lower limbs and skin regions with fatty tissue [11]. Diagnosis requires high suspicion based on clinical features and can be supported by skin biopsy [12].

Our patient presents with a typical picture of calciphylaxis; however, she was on Warfarin, and to exclude warfarin-induced ulcer, a bunch biopsy was done, and it stained positive for Von Kossa stain for calcium, which confirmed the diagnosis of calciphylaxis.

The main culprit in the pathogenesis of calciphylaxis is vascular calcification; therefore, management is mainly directed toward stopping vascular calcification [13]. Treatment should utilize a multidisciplinary approach, including control of infection, correction of electrolytes, and local management of ulcers by debridement [13].

The management of calciphylaxis involves a multifaceted approach addressing various aspects of the disease process. Key components include targeting phosphorus control to maintain levels below 5.5 mg/dL, optimizing dialysis for the clearance of uremic molecules and improving bone-mineral markers, ensuring appropriate vascular access, managing phosphate levels, addressing hyperparathyroidism, and mitigating vascular calcification. Close monitoring and coordination among healthcare providers are crucial for optimizing outcomes in patients with this challenging condition [14].

## Conclusions

Calcific uremic arteriopathy is a severe and often fatal condition in patients with ESRD. It involves calcification and narrowing of small blood vessels, leading to tissue damage and potential complications. Management of calciphylaxis is complex and typically involves a multidisciplinary approach, including wound care, pain management, calcium, and phosphorus control, vascular intervention such as wound debridement, sodium thiosulfate, nutritional support, and medications like bisphosphate, corticosteroid, and hyperbaric oxygen.
